# Case report: Rapid improvements of anorexia nervosa and probable myalgic encephalomyelitis/chronic fatigue syndrome upon metreleptin treatment during two dosing episodes

**DOI:** 10.3389/fpsyt.2023.1267495

**Published:** 2023-11-09

**Authors:** Johannes Hebebrand, Jochen Antel, Linda von Piechowski, Cordula Kiewert, Burkhard Stüve, Gertraud Gradl-Dietsch

**Affiliations:** ^1^Department of Child and Adolescent Psychiatry, Psychosomatics and Psychotherapy, Center for Translational Neuro- and Behavioral Sciences, University Hospital Essen, University of Duisburg-Essen, Essen, Germany; ^2^Department of Child and Adolescent Psychiatry, Psychosomatics and Psychotherapy, University Hospital Münster, University of Münster, Münster, Germany; ^3^Division of Pediatric Endocrinology and Diabetology, Department of Pediatrics II, University Hospital Essen, University of Duisburg-Essen, Essen, Germany; ^4^Department of Pediatric Neurology, Centre for Neuromuscular Disorders, Center for Translational Neuro- and Behavioral Sciences, University Hospital Essen, University of Duisburg-Essen, Essen, Germany; ^5^Department of Neuropediatrics, DRK Children’s Hospital, Siegen, Germany

**Keywords:** leptin, muscle, exhaustion, fatigue, depression, sleep quality

## Abstract

A comorbidity of anorexia nervosa (AN) and myalgic encephalomyelitis (ME/CSF) is uncommon. A 17 years-old male adolescent with possible onset of ME/CFS after an Epstein Barr Virus infection (EBV) and later onset of AN during a second period of weight loss was twice treated off-label with metreleptin for 15 and 11 days, respectively. As in previous cases, eating disorder specific cognitions and mood improved. Interestingly, fatigue and post-exertional muscle pain (P-EMP) improved, too. We discuss potential mechanisms. Treatment with metreleptin may prove beneficial in AN and in ME/CSF associated with substantial weight loss.

## Introduction

Human recombinant leptin (r-metHuLeptin; metreleptin) has been used off-label in single patients with anorexia nervosa [AN (1–5)]; based on an *a priori* defined hypothesis, according to which the alleviation of hypoleptinemia induces beneficial cognitive, emotional and behavioral changes (6). Metreleptin treatment for six to 24 days was associated with a rapid onset and reduction of starvation related symptoms including preoccupation with food, fatigue, depression, inner tension, rigidity, urge to move and hyperactivity. Limited evidence suggests that metreleptin treatment may also have antidepressant effects in other conditions associated with inborn or acquired leptin deficiency (5, 7, 8).

Myalgic encephalomyelitis/chronic fatigue syndrome (ME/CSF) is one of the post-acute infection syndromes (9). An infection precedes the development of ME/CSF in approximately 75% of cases (9). The post-acute sequelae of SARS-CoV-2 infection (“long COVID”) share several features with ME/CSF (9). A decreased ability of the immune system to control/eliminate EBV infection has been hypothesized to be responsible for ME/CSF, celiac disease, multiple sclerosis, rheumatoid arthritis, and other autoimmune diseases (10).

Fatigue, post-exertional malaise, neurocognitive problems, and unrefreshing sleep are common in ME/CSF (11). Additional symptoms have been included in different classifications. The International Consensus Criteria (ICC) for myalgic encephalomyelitis lists symptoms for four domains including post-exertional neuroimmune exhaustion, neurological impairments, immune/gastro-intestinal/genitourinary impairments, and energy production and ion transport impairments (12). Both weight gain and weight loss may be associated, the latter requiring the exclusion of underlying morbidity (13, 14). Differential diagnoses for longer standing fatigue include celiac disease, major depression and eating disorders. However, if the respective disease does not largely account for the symptoms characteristic of ME/CSF, these disorders may represent co-morbidities according to a recent expert consensus (13).

## Case description

We describe a currently 18 years old male who had developed both probable ME/CSF and AN after an Epstein Barr virus (EBV) infection. Family history was uneventful apart from a major depressive disorder (MDD) of the maternal grandfather. The patient had discrete separation anxiety upon entrance to kindergarten and speech therapy for dyslalia. During childhood, otitis media was treated repeatedly with antibiotics, a salmonella infection required a 5 days hospitalization. The achievement-oriented youth played a ball game competitively at the national level. After otherwise normal somatic, emotional, social and cognitive/academic development the patient fell ill at age 15 years and 8 months with an EBV infection (EBV IGM 60 U/mL, IgG 97 U/mL 1 month after initial symptoms; cytomegalovirus IGM and IGG negative) with splenomegaly and enlarged lymph nodes. His physical condition deteriorated entailing repeated brief hospitalizations. Weakness, loss of appetite and constipation were prominent.

### Diagnostic assessment and therapeutic interventions

After exclusion of systemic infections, hemato-oncologic disorders (bone marrow biopsy), autoimmune, endocrine and neuromuscular disorders [whole body magnetic resonance imaging, normal creatinine kinase serum levels (CK), metabolic workup, muscle biopsy and genetic testing] CFS was diagnosed. The patient was intermittently able to walk a few steps only with physical exertion induced muscle pain in the upper arms and legs and post-exertional malaise which could persist the following day. Weakness and tingling paresthesia as well as episodes of headaches and dizziness were noted 3 months after onset of the infection. After 6 months nerve pain appeared at the right neck triangle and celiac disease (Marsh type 3b) was diagnosed via biopsy.

The introduction of a gluten free diet led to an improvement of constipation; a second biopsy conducted 5 months later revealed normal results. Weight loss amounted to 15 kg 7 months after onset of EBV infection (recalled premorbid weight 69 kg), after which a body weight of around 53–55 kg was maintained for approximately 12 months (Supplementary Figure S1A).

He was admitted for inpatient adolescent psychiatric treatment at age 17 years and 8 months his body weight had dropped to 42.6 kg (BMI 12.45 kg/m^2^; Supplementary Figure S1B). Fear of weight gain, body image disturbances, and an urge to move were prominent leading to the diagnosis of AN according to DSM-5 criteria (15). Somatic and laboratory findings were compatible with AN and included pericardial effusion, increased echogenicity of liver and kidneys, low T3, thrombocytopenia, reduced gonadotropin and testosterone levels, elevations of liver enzymes, cholinesterase, alpha-amylase, lipase, basal cortisol, CK and ferretin.

Multimodal treatment according to German treatment guidelines (16) led to weight gain (≈1 kg/week; Supplementary Figure S1B). Multimodal treatment of AN entails psychotherapy (16), nutritional management and in individual cases pharmacotherapy (16). During realimentation mood deteriorated; after 14 weeks and at a BMI of 15.81 kg/m^2^ the patient became suicidal and had to be transferred intermittently to the secure ward. Complaints included: sense of pronounced hopelessness, complete lack of perspective, poor sleep, fatigue, constant preoccupation with food and body weight, and distorted body image. The urge to move resulted in physical activity which entailed intensive muscle pain. Based on previous improvements of mood and sleep upon off-label treatment with metreleptin a dosing period of 15 days was initiated after the patient and his parents provided written informed consent.

### First dosing period

Metreleptin was applied subcutaneously at 9:00 am for 15 consecutive days. Doses ranged from 3 to 6 mg/days. A COVID-19 infection of another patient in the ward required immediate discharge on day 5 according to hygiene regulations; both the patient and his parents agreed to proceed with outpatient dosing despite uncertainty as to potential effects of metreleptin on the severity of a COVID-19 infection. The patient himself was not infected. Inpatient treatment was reinitiated on *d* + 3.

Visual analogue scales (VAS; morning and evening scores; items ranked from 1 to 10) were used to track emotions and cognitions daily (1–3); the immediate discharge on day 5 entailed that VAS self-ratings were not obtained on the evening of day 5 and the morning of day 8, both morning and evening ratings are missing for day 6 and day 7. Self-ratings based on the German versions of Beck Depression Inventory-II (17) and Eating Disorder Inventory-2 (18) were obtained prior to, during and after the dosing period. At baseline, the German Chronic Fatigue Syndrome Questionnaire [CFSQ (19)], was employed. Serum leptin levels (ELISA) were measured at 9 am; for estimation of leptin level percentile adjusted for sex, Tanner stage and BMI we referred to the user’s manual (20, 21).

The patient was concomitantly treated with olanzapine 7.5 mg (3 × 2.5 mg) daily. Psychoeducation focused on expected treatment effects, the presumed underlying mechanism (alleviation of a hormone deficiency), and the necessity to use the boost in motivation to gain weight in order to enable sufficient endogenous leptin secretion.

### Second dosing period

Metreleptin (3 mg/days) was applied subcutaneously at 9:00 am for 11 consecutive days. Again, the patient was concomitantly treated with 3 × 2.5 mg olanzapine daily. Psychotherapeutic support was provided throughout the hospital stay, including both dosing periods.

## Outcome and follow-up

### First dosing period

**Figure 1:**
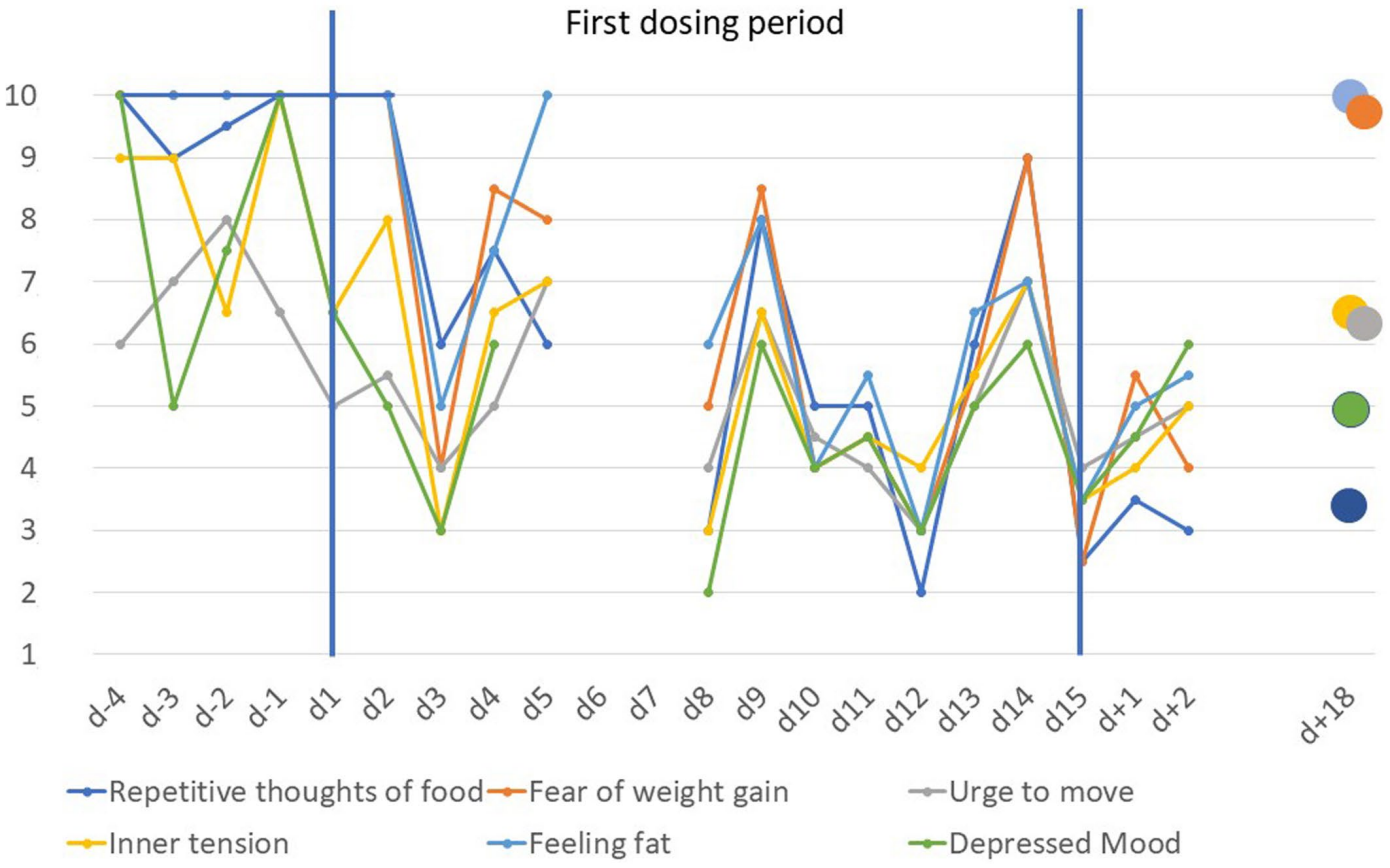
Visual analogue scale scores scaled from 1 to 10 for anorexia nervosa related items prior to, during and after the first dosing period (*d* − 4 to *d* + 18). Scores are based on the means of ratings in the morning and evening except for day 5 (morning rating only) and day 8 (evening rating only); the immediate discharge from inpatient treatment on day 5 also led to missing data for day 6 and day 7.

Similar to previous findings (1–3), daily means of VAS items revealed reductions in repetitive thoughts of food, fear of weight gain, feeling fat, inner tension, depressed mood, and urge to move (Figure 1). At *d* + 18 scores of the items feeling fat and fear of weight gain had rebounded completely.

**Figure 2:**
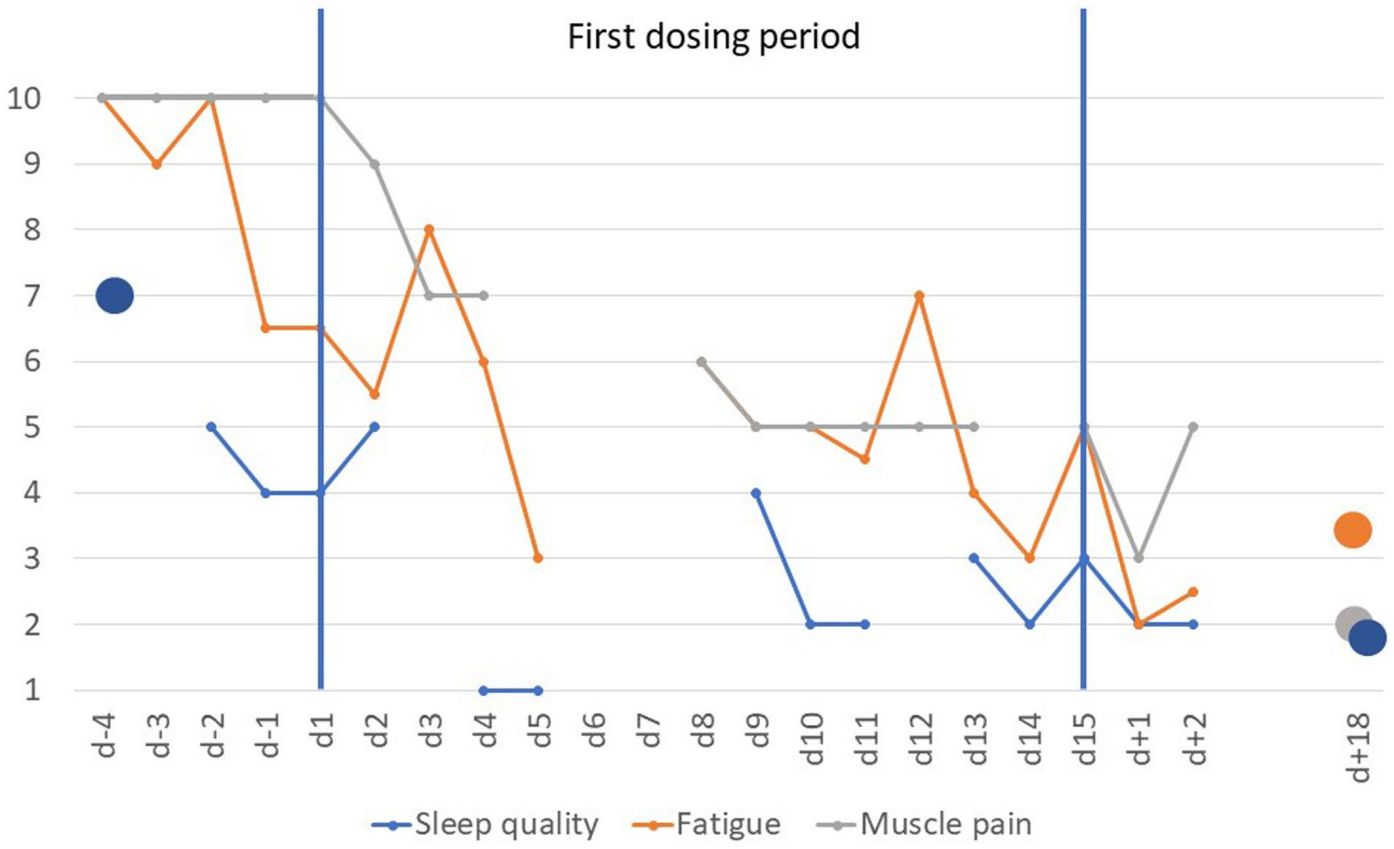
Visual analogue scale scores scaled from 1 to 10 for items fatigue, sleep quality and muscle pain prior to, during and after the first dosing period (*d* − 4 to *d* + 18). Scores for fatigue are based on the means of ratings obtained mornings and evenings except for day 5 (morning rating only) and day 8 (evening rating only); sleep quality was rated once daily (1 = very good; VAS item was not scored on *d* − 3, day 3 and day 12). In contrast, only evening scores for muscle pain were used (morning ratings during the dosing period skewed between 2 and 4); missing data for day 5 to day 7 (see Methods).

The baseline responses provided in the CFSQ were compatible with a diagnosis of CFS. VAS items fatigue and muscle pain (evening ratings only) dropped rapidly after initiation of dosing (Figure 2). Sleep quality (scores based mainly on morning ratings) improved after initiation of dosing (Figure 2). In contrast, tiredness showed no systematic trend with mean scores ranging from 3 to 7. The patient reported being able to sleep through the night or having to void only once a night upon the end of the dosing period (approximately four awakenings at baseline).

Self-rated depression BDI-II score dropped from 42 (*d* − 3) to 25 (day 4) and 20 (*d* + 3) to reach the low nadir of 16 on *d* + 13 (*d* + 18: 23). The raw score of the EDI-2 dropped from 377 (*d* − 3; 18.3.22) to 331 (*d* + 17; >99th percentile at both time points).

During the initial 11 weeks of inpatient treatment serum leptin levels proved undetectable (see Supplementary Figure S1B). The level of 1.7 ng/mL prior to initial dosing (day 1; BMI 16.22 kg/m^2^) exceeded the 99th percentile of leptin levels adjusted for sex, Tanner stage and BMI.

At follow up *d* + 79, muscle pain continued to be diminished. The patient reported no pain upon normal walking or marching. However, upon jogging muscle pain rapidly set in. Fatigue, too, was not viewed as a complaint any longer. Concentration problems persisted; the patient expressed frustration due to memory problems in school (attendance of 2 h daily only). He had reinitiated social contacts. Mood swings represented an additional complaint. Overall, his condition, including eating disorder related symptoms had further improved in comparison to discharge from inpatient treatment (*d* + 2). BMI increased over the course of the treatment (Supplementary Figure S1B_2).

### Second dosing period

**Figure 3:**
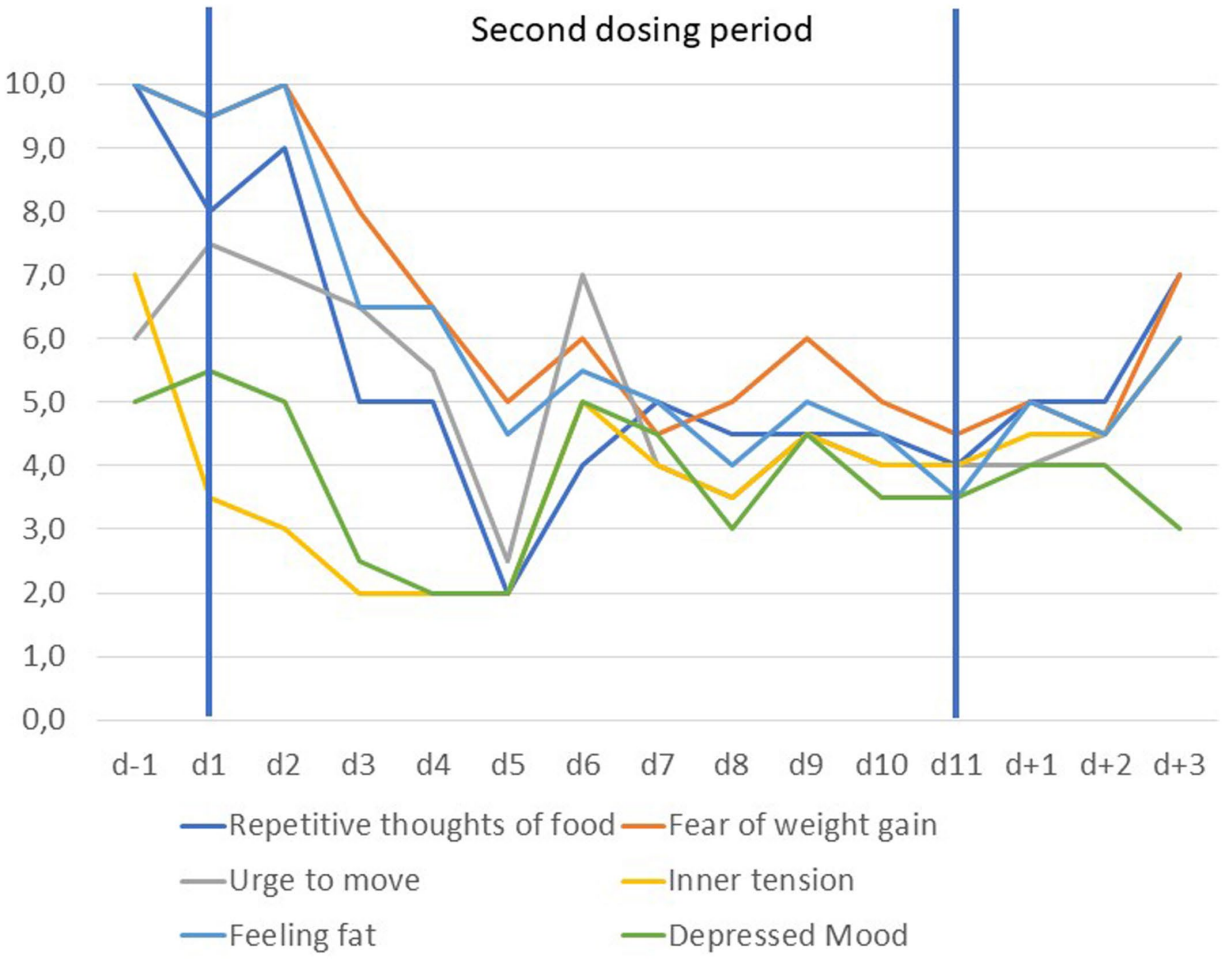
Visual analogue scale scores scaled from 1 to 10 for anorexia nervosa related items prior to, during and after the second dosing period (*d* − 1 to *d* + 3). Scores are based on the means of ratings in the morning and evening.

**Figure 4:**
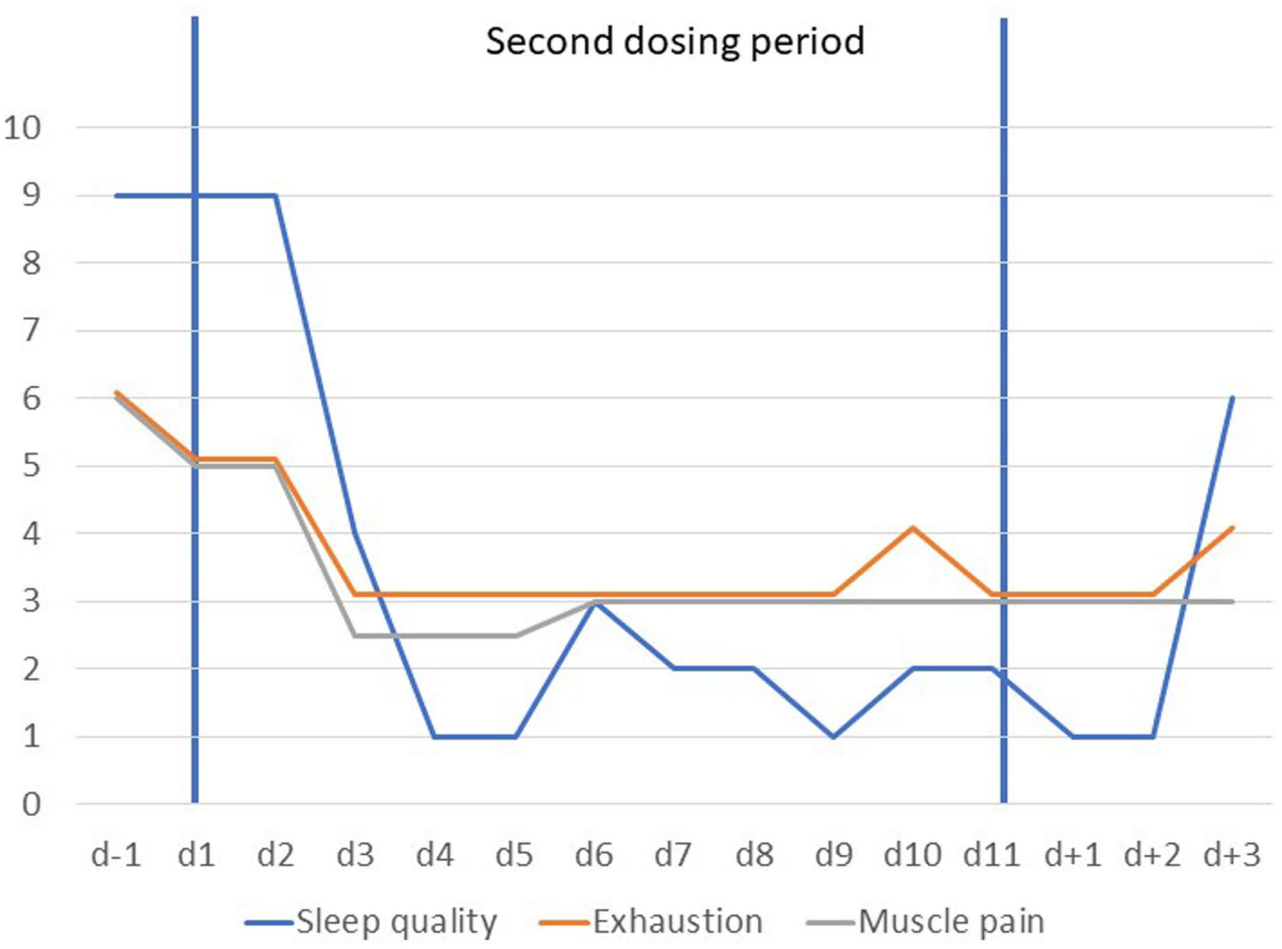
Visual analogue scale scores scaled from 1 to 10 for items tiredness, sleep quality and muscle pain prior to, during and after the second dosing period (*d* − 1 to *d* + 3). Scores for fatigue are based on the means of ratings obtained mornings and evenings; sleep quality was rated once daily (1 = very good).

Five months after the first dosing, his condition again deteriorated. The patient reported fatigue, increasing muscle pain following exercise and insomnia. He asked for a second dosing period which appeared justified based on an assessment of somatic and psychiatric parameters and the initially positive response. After initiation of dosing all VAS items dropped rapidly during the first 5 days (see Figure 3). An unexplained rebound occurred at day 6 followed by a slight further reduction of the respective VAS items. A slight rebound seemingly occurred from *d* + 2 onwards for all items except depressed mood. Sleep quality, fatigue and muscle pain improved again with a fast onset (see Figure 4). Self-rated depression BDI-II score dropped from 25 (*d* − 1) to 8 (day 9). BMI dropped from 18.8 kg/m^2^ by 0.4 kg/m^2^ during the dosing period but returned to the same value (18.8 kg/m^2^) at the end of the treatment period (Supplementary Figure S1C).

No adverse events were reported in either the first or second dosing period.

### Long term follow up

After discharge from inpatient treatment the patient continued to struggle with AN related symptoms, which slowly improved. The BMI development over the whole course of the treatment is shown in Supplementary Figure S1D. At *d* + 476 (1st dosing episode) he is working full-time as an apprentice in a bakery; eating disorder specific cognitions persist at a subclinical level and muscle pain was no longer reported. The current BMI is 19.11 kg/m^2^. He recently began sports activities again. A relapse did not occur, but cannot be excluded in light of the low BMI.

## Discussion

This case report is remarkable for the rapid improvement of symptoms of both AN and probable ME/CSF upon two short-term metreleptin treatments. The positive changes set in shortly after the beginnings of both dosing episodes, but partly faded after the end these episodes, which is also reflected in the high baseline values of some VAS items at the beginning of the second dosing. We cautiously refer to probable ME/CSF, because we focused our clinical assessment on P-EMP; post-exertional malaise was not systematically assessed. We also did not evaluate the ME/CSF symptomatology with questionnaires based on the Canadian Consensus Criteria or ICC criteria (12, 22). Finally, the overlap of symptoms of ME/CSF and AN warrant caution with respect to their diagnostic differentiation.

In our patient an Epstein–Barr virus infection initially resulted in weight loss, fatigue and P-EMP and malaise, for which no underlying medical condition was detected. Concentration and memory problems had plagued the patient from the beginning on. The initial weight loss of approximately 15 kg was substantial and plateaued for 12 months, after which further 11.5 kg were lost resulting in an exceedingly low referral BMI of 12.45 kg/m^2^. At this time point, weight phobia, body image disturbances, fatigue and evening muscle pain dominated the clinical symptomatology, which in combination with the severe underweight led to the DSM-5 diagnosis of AN in addition to preexisting probable ME/CSF.

Weight loss can occur with EBV infection (23) and ME/CSF (13). According to a Latvian study, approximately 25% of ME/CSF patients experience weight loss (24), which in single cases can be severe (25). Single case reports have reported the development of AN after EBV-infection (26) or glandular fever’-like illnesses (27). A comorbidity between ME/CSF and AN has been observed previously (28, 29); in our patient, symptoms of ME/CSF set in approximately 12 to 18 months prior to the emergence of AN specific cognitions.

Triggered by a remark from one of our reviewers, we briefly discuss a potential immune system involvement as an overlapping pathophysiological hypothesis for the simultaneous occurrence of probable ME/CFS and AN. Immune dysregulation in ME/CFS is common and a subset of patients with ME/CFS may have an autoimmune etiology (30). Some studies have provided evidence for severe metabolic disturbances potentially mediated by serum autoantibodies in ME/CFS (30). In AN, immunological dysfunctions and reciprocal associations between this eating disorder and autoimmune diseases suggest a role of autoimmunity in its pathogenesis (31). The microbiome may represent a potential link; microbiota affect the immune system (31), disrupt gut-brain communication, and may trigger eating disorders including AN (32). Disrupted T cell tolerance and autoantibodies were observed in AN (31) and there is also evidence for immuno-metabolic dysfunction in T cell and natural killer (NK) cell populations in ME/CFS (33). Speculatively, alterations of the immune response and the production of autoantibodies might be a common pathogenic substrate for both conditions.

Apart from fatigue, early onset P-EMP was pronounced in our patient; post-exertional malaise persisted until the next day. Within weeks after onset of the EBV infection, the patient complained of up to five nightly bladder voidings consistent with a genitourinary symptom of ME/CSF (12). In accordance with symptoms observed in ME/CSF, the patient also experienced episodes of headache. In addition, celiac disease became manifest 6 months after onset of infectious mononucleosis, which may fit into immune gastro-intestinal symptoms of ME/CSF (12). Importantly, introduction of a gluten free diet had no effect on symptoms of ME/CSF but led to normal results of a second duodenal biopsy 5 months later.

Upon hospitalization for treatment of AN 2.0 years after onset of EBV-infection, the longstanding P-EMP interfered with the urge to move, which is a common symptom of AN, as is hyperactivity (34, 35). While P-EMP and malaise are not complaints of patients with AN, single studies have reported muscle pain in three rather severely underweight patients with longstanding AN (36). Over-exercising occurred in two of these patients; coexistent fatigability occurred only for a matter of weeks. Typically, patients with AN experience hyperactivity in combination with tiredness and exhaustion (34, 35). Muscle soreness and cramps were noted in participants of the Minnesota Starvation Experiment (37).

During the initial dosing period the patient received 3 to 6 mg per day. The parents had noticed strong changes during the dosing days with 6 mg. It is unclear if more pronounced improvements would have been associated with continuous application of 6 mg and/or a longer dosing period. Mood improvement (VAS) in the current case was somewhat less pronounced than in other patients; nevertheless, BDI-II scores were approximately halved after the dosing period. For an intermittent period of time mood improved around noon. Mood swings were rated high throughout dosing. Notably, these changes occurred despite an exceedingly high baseline serum leptin level adjusted for sex, Tanner stage and BMI, which likely reflects prior weight gain (38).

The improvements in sleep quality, depression, and eating disorder cognitions have been reported previously in five patients with AN (1–3). In addition, an improvement in executive functioning and in particular short-term memory and the ability to concentrate occurred in some of these patients. Evidently, poor sleep and cognitive difficulties occur in ME/CFS, too. Rodent studies indicative of a role of leptin in sleep, mood and cognition (5, 39) and the rapid amelioration upon treatment suggest potentially overlapping mechanisms.

In the following we focus on two cardinal symptoms of ME/CSF, namely fatigue and P-EMP. The mechanism underlying fatigue in ME/CFS is unclear. We discuss possible explanations for the observed improvements.

- Leptin is assumed to contribute to the inflammatory state found in obesity that predisposes to metabolic syndrome, type 2 diabetes mellitus, and cardiovascular disease. However, leptin is also an important mediator of the immunosuppressive state in undernutrition (40).
- The improved sleep quality and/or improved mood may entail a reduction of the subjective feeling of fatigue. Leptin has strong effects on both sleep and behaviors suggestive of depression in *ob*/*ob* mice (5, 39). In patients with AN, metreleptin treatment was associated with oversleeping (1, 39). The improved sleep and daytime tiredness were experienced as wholesome and healthy, they were not associated with a feeling of a hangover upon awakening. Analogously, the mood improvement may entail a reduction of experienced tiredness/fatigue.
- Both the leptin and leptin receptor genes are expressed in muscle (41). Increments in circulating leptin decrease the expression of the leptin gene in adipose tissue, while inducing an increase in skeletal muscle, indicating that leptin secretion can function as a vehicle of “cross-talk” between both tissues with tissue-specific modulation of the leptin signal (42). Absence of leptin signaling in mouse skeletal muscle induced fiber hypoplasia and muscle atrophy (43). Leptin receptors in skeletal muscle and their signaling cascade have been shown to be up-regulated in response to a severe energy deficit (44). Skeletal muscle capillarity is down-regulated in leptin deficient states (45). Muscle pain is generated via nociceptors by adenosine triphosphate (ATP) via the P2X3 receptor and H^+^ ions via binding to the receptor molecules transient receptor potential vanilloid 1 (TRPV1) and acid-sensing ion channels (46). In our patient, evening ratings of muscle pain dropped substantially during both dosing periods. After cessation of dosing, the patient reported no longer experiencing pain upon walking; this improvement persisted and allowed the patient to engage in more physical activity. However, pain reappeared quickly upon jogging. Prior to dosing, he suffered from a strong urge to move, which if pursued, entailed strong muscle pain (ranked maximal in respective item of VAS).

Leptin in ME/CSF has not been a major focus of research. A review has been registered with PROSPERO with the aim of examining circulating levels of leptin in patients with ME/CFS or fibromyalgia syndrome and healthy controls (47). According to a non-peer reviewed publication, no convincing evidence indicates that leptin levels are higher in cases than controls: six studies found higher, four studies no differences and two lower levels in cases as compared to controls (48). Leptin and an additional 16 mostly pro-inflammatory cytokines revealed a statistically significant upward linear trend that correlated with ME/CFS severity (49).

In conclusion, in addition to the need of randomized controlled trials for the use of metreleptin in AN, this case report should lead to an increased awareness of ME/CSF patients with pronounced weight loss. A comorbid diagnosis of AN may apply to some of these patients. Metreleptin may have beneficial effects in those patients with ME/CFS who have lost a substantial amount of weight entailing low serum leptin levels.

## Limitations

The concomitant treatment with olanzapine and psychotherapy during both dosing episodes represents a limitation of our case study. In particular, we cannot exclude a drug interaction. However, several of the observed improvements were similar to those observed in previous case reports [AN (1–5)]; irrespective of concomitant medication. In particular, olanzapine is known to impact sleep (50). Indeed, the patient reported increased tiredness after starting olanzapine, but poor sleep quality (frequent awakenings) persisted. As in other cases (39), improved sleep quality was only reported in the context of metreleptin administration.

## Data availability statement

All directly sharable data are supplied with the Supplementary material.

## Ethics statement

Ethical approval was not required for the study involving human samples in accordance with the local legislation and institutional requirements because this is a case report no research intervention was conducted. Written informed consent for participation in this study was provided by the participants’ legal guardians/next of kin. Written informed consent was obtained from the individual(s), and minor(s)’ legal guardian/next of kin, for the publication of any potentially identifiable images or data included in this article. Written informed consent was obtained from the participant/patient(s) for the publication of this case report.

## Author contributions

JH: Conceptualization, Writing – original draft, Writing – review & editing. JA: Writing – review & editing, Visualization. LP: Data curation, Investigation, Writing – review & editing. CK: Data curation, Investigation, Writing – review & editing. BS: Data curation, Investigation, Writing – review & editing. GG-D: Data curation, Investigation, Writing – review & editing.

## Supplementary material

SUPPLEMENTARY FIGURE S1A Course of BMI (kg/m^2^) over the initial 23 months after EBV infection starting with the premorbid value of 20.1 kg/m^2^; no measurements available for months 19 to 23; child and adolescent psychiatric inpatient treatment occurred as of month 24 (see Supplementary Figure S1B).

SUPPLEMENTARY FIGURE S1B BMI values prior to, during and after the first 15-day dosing period (marked by the two vertical lines) and serum leptin levels during the 24-week long treatment period. The dosing period was during weeks 15 and 17. Leptin levels weeks 1 to 11 were <0.1 ng/ml.

SUPPLEMENTARY FIGURE S1B_2 BMI changes seven days prior, during and after Dosing Period 1.

SUPPLEMENTARY FIGURE S1C BMI changes seven days prior, during and after Dosing Period 2.

SUPPLEMENTARY FIGURE S1D BMI over the course of treatment; dosing periods marked by squares.

## References

[1] Milos G Antel J Kaufmann LK Barth N Koller A Tan S. Short-term metreleptin treatment of patients with anorexia nervosa: rapid on-set of beneficial cognitive, emotional, and behavioral effects. Transl Psychiatry. (2020) 10:303. doi: 10.1038/s41398-020-00977-1 32855384 PMC7453199

[2] Antel J Tan S Grabler M Ludwig C Lohkemper D Brandenburg T. Rapid amelioration of anorexia nervosa in a male adolescent during metreleptin treatment including recovery from hypogonadotropic hypogonadism. Eur Child Adolesc Psychiatry. (2021) 31:1573–9. doi: 10.1007/s00787-021-01778-7 33966118 PMC8106547

[3] Gradl-Dietsch G Bell R Tschöpe F Milos G Wabitsch M Antel J. Rapid emergence of appetite and hunger resulting in weight gain and improvement of eating disorder symptomatology during and after short-term off-label metreleptin treatment of a patient with anorexia nervosa. Obes Facts. (2023) 16:99–107. doi: 10.1159/000527386, PMID: 36349765 PMC9889726

[4] Hebebrand J Hinney A Antel J. Could leptin substitution therapy potentially terminate entrapment in anorexia nervosa? Nat Rev Endocrinol. (2023) 19:435–6. doi: 10.1038/s41574-023-00863-y, PMID: 37316581

[5] Hebebrand J Hildebrandt T Schlögl H Seitz J Denecke S Vieira D. The role of hypoleptinemia in the psychological and behavioral adaptation to starvation: implications for anorexia nervosa. Neurosci Biobehav Rev. (2022) 141:104807. doi: 10.1016/j.neubiorev.2022.104807, PMID: 35931221

[6] Hebebrand J Milos G Wabitsch M Teufel M Fuhrer D Buhlmeier J. Clinical trials required to assess potential benefits and side effects of treatment of patients with anorexia nervosa with recombinant human leptin. Front Psychol. (2019) 10:769. doi: 10.3389/fpsyg.2019.00769 31156489 PMC6533856

[7] Vieira D Antel J Peters T Miehle K Stumvoll M Hebebrand J. Suggestive evidence for an antidepressant effect of metreleptin treatment in patients with lipodystrophy; under revision. Obes Facts. (2022) 15:685–93. doi: 10.1159/000526357, PMID: 36037795 PMC9669995

[8] von Schnurbein J Remy M Brandt S Manzoor J Kohlsdorf K Mahmood S. Positive effect of leptin substitution on mood and behaviour in patients with congenital leptin deficiency. Pediatr Obes. (2023):e13057. doi: 10.1111/ijpo.13057, PMID: 37226403

[9] Choutka J Jansari V Hornig M Iwasaki A. Unexplained post-acute infection syndromes. Nat Med. (2022) 28:911–23. doi: 10.1038/s41591-022-01810-6, PMID: 35585196

[10] Ruiz-Pablos M Paiva B Montero-Mateo R Garcia N Zabaleta A. Epstein–Barr virus and the origin of myalgic encephalomyelitis or chronic fatigue syndrome. Front Immunol. (2021) 12:656797. doi: 10.3389/fimmu.2021.656797, PMID: 34867935 PMC8634673

[11] Jason LA Evans M Brown A Sunnquist M Newton JL. Chronic fatigue syndrome versus sudden onset myalgic encephalomyelitis. J Prev Interv Community. (2015) 43:62–77. doi: 10.1080/10852352.2014.973233 25584529 PMC4295655

[12] Carruthers BM van de Sande MI De Meirleir KL Klimas NG Broderick G Mitchell T. Myalgic encephalomyelitis: international consensus criteria. J Intern Med. (2011) 270:327–38. doi: 10.1111/j.1365-2796.2011.02428.x, PMID: 21777306 PMC3427890

[13] Nacul L Authier FJ Scheibenbogen C Lorusso L Helland IB Martin JA. European network on myalgic encephalomyelitis/chronic fatigue syndrome (EUROMENE): expert consensus on the diagnosis, service provision, and care of people with ME/CFS in Europe. Medicina. (2021) 57:510. doi: 10.3390/medicina57050510, PMID: 34069603 PMC8161074

[14] Trabal J Leyes P Fernández-Solá J Forga M Fernández-Huerta J. Patterns of food avoidance in chronic fatigue syndrome: is there a case for dietary recommendations? Nutr Hosp. (2012) 27:659–62. doi: 10.1590/S0212-16112012000200046, PMID: 22732998

[15] American Psychiatric Association. DSM-5: diagnostic and statistical manual of mental disorders. Washington, DC: American Psychiatric Association (2013).

[16] Resmark G Herpertz S Herpertz-Dahlmann B Zeeck A. Treatment of anorexia nervosa-new evidence-based guidelines. J Clin Med. (2019) 8:153. doi: 10.3390/jcm8020153, PMID: 30700054 PMC6406277

[17] Hautzinger M Keller F Kühner C. BDI-II: Beck depressions inventar revision-manual. Frankfurt: Harcourt Test Services (2006).

[18] Thiel A Jacobi C Horstmann S Paul T Nutzinger DO Schüssler G. A German version of the eating disorder inventory EDI-2. Psychother Psychosom Med Psychol. (1997) 47:365–76. PMID: 9411465

[19] Nater UM Fischer S Latanzio S Ruoss D Gaab J. FFSS – Fragebogen zur Erfassung funktioneller somatischer Syndrome. Verhaltenstherapie. (2011) 21:263–5. doi: 10.1159/000333298

[20] IBL-International, Leptin ELISA, (2020). Available at: https://md53001_ifu_eu_en_leptin_elisa_v003-01_2022-12_sym9.pdf (ibl-international.com).

[21] Blum WF Juul A. Reference ranges of serum leptin, in leptin-the voice of adipose tissue. Heidelberg: Johann Ambrosius Verlag (1997).

[22] Carruthers BM Jain AK De Meirleir KL Peterson DL Klimas NG Lerner AM. Myalgic encephalomyelitis/chronic fatigue syndrome: clinical working case definition, diagnostic and treatment protocols. J Chronic Fatigue Syndr. (2003) 11:7–115. doi: 10.1300/J092v11n01_02

[23] Tynell E Aurelius E Brandell A Julander I Wood M Yao QY. Acyclovir and prednisolone treatment of acute infectious mononucleosis: a multicenter, double-blind, placebo-controlled study. J Infect Dis. (1996) 174:324–31. doi: 10.1093/infdis/174.2.324, PMID: 8699062

[24] Krumina A Vecvagare K Svirskis S Gravelsina S Nora-Krukle Z Gintere S. Clinical profile and aspects of differential diagnosis in patients with ME/CFS from Latvia. Medicina. (2021) 57:958. doi: 10.3390/medicina57090958, PMID: 34577881 PMC8467618

[25] Dafoe W. Extremely severe ME/CFS-A personal account. Healthcare. (2021) 9:504. doi: 10.3390/healthcare9050504, PMID: 33925566 PMC8145314

[26] Simon MW. Anorexia and failure to grow associated with Epstein–Barr virus infection. J Ky Med Assoc. (1998) 96:13–5. PMID: 9470311

[27] Park RJ Lawrie SM Freeman CP. Post-viral onset of anorexia nervosa. Br J Psychiatry. (1995) 166:386–9. doi: 10.1192/bjp.166.3.386, PMID: 7788133

[28] Fisher M Krilov LR Ovadia M. Chronic fatigue syndrome and eating disorders: concurrence or coincidence? Int J Adolesc Med Health. (2002) 14:307–16. doi: 10.1515/ijamh.2002.14.4.307, PMID: 12613112

[29] Nater UM Lin JM Maloney EM Jones JF Tian H Boneva RS. Psychiatric comorbidity in persons with chronic fatigue syndrome identified from the Georgia population. Psychosom Med. (2009) 71:557–65. doi: 10.1097/PSY.0b013e31819ea179, PMID: 19414619

[30] Sotzny F Blanco J Capelli E Castro-Marrero J Steiner S Murovska M. Myalgic encephalomyelitis/chronic fatigue syndrome—evidence for an autoimmune disease. Autoimmun Rev. (2018) 17:601–9. doi: 10.1016/j.autrev.2018.01.009, PMID: 29635081

[31] Sirufo MM Magnanimi LM Ginaldi L De Martinis M. Anorexia nervosa and autoimmune comorbidities: a bidirectional route? CNS Neurosci Ther. (2022) 28:1921–9. doi: 10.1111/cns.13953, PMID: 36114699 PMC9627382

[32] Seitz J Trinh S Herpertz-Dahlmann B. The microbiome and eating disorders. Psychiatr Clin North Am. (2019) 42:93–103. doi: 10.1016/j.psc.2018.10.004 30704642

[33] Maya J. Surveying the metabolic and dysfunctional profiles of T cells and NK cells in myalgic encephalomyelitis/chronic fatigue syndrome. Int J Mol Sci. (2023) 24:11937. doi: 10.3390/ijms241511937, PMID: 37569313 PMC10418326

[34] Casper RC. Might starvation-induced adaptations in muscle mass, muscle morphology and muscle function contribute to the increased urge for movement and to spontaneous physical activity in anorexia nervosa? Nutrients. (2020) 12:2060. doi: 10.3390/nu12072060, PMID: 32664448 PMC7400818

[35] Casper RC Voderholzer U Naab S Schlegl S. Increased urge for movement, physical and mental restlessness, fundamental symptoms of restricting anorexia nervosa? Brain Behav. (2020) 10:e01556. doi: 10.1002/brb3.1556, PMID: 32017454 PMC7066368

[36] McLoughlin DM Spargo E Wassif WS Newham DJ Peters TJ Lantos PL. Structural and functional changes in skeletal muscle in anorexia nervosa. Acta Neuropathol. (1998) 95:632–40. doi: 10.1007/s004010050850, PMID: 9650756

[37] Keys A Brozek J Henschel A Mickelsen O Taylor HL. The biology of human starvation University of Minnesota Press (1950).

[38] Hebebrand J Blum WF Barth N Coners H Englaro P Juul A. Leptin levels in patients with anorexia nervosa are reduced in the acute stage and elevated upon short-term weight restoration. Mol Psychiatry. (1997) 2:330–4. doi: 10.1038/sj.mp.4000282, PMID: 9246674

[39] Hebebrand J Denecke S Antel J. The role of leptin in rodent and human sleep: a transdiagnostic approach with a particular focus on anorexia nervosa. Neurosci Biobehav Rev. (2023) 149:105164. doi: 10.1016/j.neubiorev.2023.105164 37031924

[40] Perez-Perez A Vilarino-Garcia T Fernandez-Riejos P Martin-Gonzalez J Segura-Egea JJ Sanchez-Margalet V. Role of leptin as a link between metabolism and the immune system. Cytokine Growth Factor Rev. (2017) 35:71–84. doi: 10.1016/j.cytogfr.2017.03.001, PMID: 28285098

[41] Wang J Liu R Hawkins M Barzilai N Rossetti L. A nutrient-sensing pathway regulates leptin gene expression in muscle and fat. Nature. (1998) 393:684–8. doi: 10.1038/31474, PMID: 9641678

[42] Wang J Liu R Liu L Chowdhury R Barzilai N Tan J. The effect of leptin on Lep expression is tissue-specific and nutritionally regulated. Nat Med. (1999) 5:895–9. doi: 10.1038/11335, PMID: 10426312

[43] Arounleut P Bowser M Upadhyay S Shi XM Fulzele S Johnson MH. Absence of functional leptin receptor isoforms in the POUND (Lepr (db/lb)) mouse is associated with muscle atrophy and altered myoblast proliferation and differentiation. PLoS One. (2013) 8:e72330. doi: 10.1371/journal.pone.0072330, PMID: 23967295 PMC3743798

[44] Perez-Suarez I Ponce-González JG de La Calle-Herrero J Losa-Reyna J Martin-Rincon M Morales-Alamo D. Severe energy deficit upregulates leptin receptors, leptin signaling, and PTP1B in human skeletal muscle. J Appl Physiol. (1985) 123:1276–87. doi: 10.1152/japplphysiol.00454.2017 28729389

[45] Nwadozi E Ng A Stromberg A Liu HY Olsson K Gustafsson T. Leptin is a physiological regulator of skeletal muscle angiogenesis and is locally produced by PDGFRα and PDGFRβ expressing perivascular cells. Angiogenesis. (2019) 22:103–15. doi: 10.1007/s10456-018-9641-6, PMID: 30121753

[46] Mense S. Muscle pain: mechanisms and clinical significance. Dtsch Arztebl Int. (2008) 105:214–9. doi: 10.3238/artzebl.2008.0214, PMID: 19629211 PMC2696782

[47] Musker M McArthur A Munn Z Wong ML. Circulating leptin levels in patients with myalgic encephalomyelitis, chronic fatigue syndrome or fibromyalgia: a systematic review protocol. JBI Evid Synth. (2021) 19:695–701. doi: 10.11124/JBIES-20-00125, PMID: 33136710

[48] Musker M. Circulating leptin levels in patients with myalgic encephalomyelitis chronic fatigue syndrome and/or fibromyalgia: a systematic review. University of Adelaide (2021). 10.11124/JBIES-20-00125 33136710

[49] Montoya JG Holmes TH Anderson JN Maecker HT Rosenberg-Hasson Y Valencia IJ. Cytokine signature associated with disease severity in chronic fatigue syndrome patients. Proc Natl Acad Sci U S A. (2017) 114:E7150–e7158. doi: 10.1073/pnas.1710519114, PMID: 28760971 PMC5576836

[50] Boachie A Goldfield GS Spettigue W. Olanzapine use as an adjunctive treatment for hospitalized children with anorexia nervosa: case reports. Int J Eat Disord. (2003) 33:98–103. doi: 10.1002/eat.10115, PMID: 12474205

