# Supplementary figures and images for: Case report: Rapid improvements of anorexia nervosa and probable myalgic encephalomyelitis/chronic fatigue syndrome upon metreleptin treatment during two dosing episodes
Source: Front Psychiatry. 2023 Nov 9;14:1267495. doi: 10.3389/fpsyt.2023.1267495 (PMC10666640; doi:10.3389/fpsyt.2023.1267495)

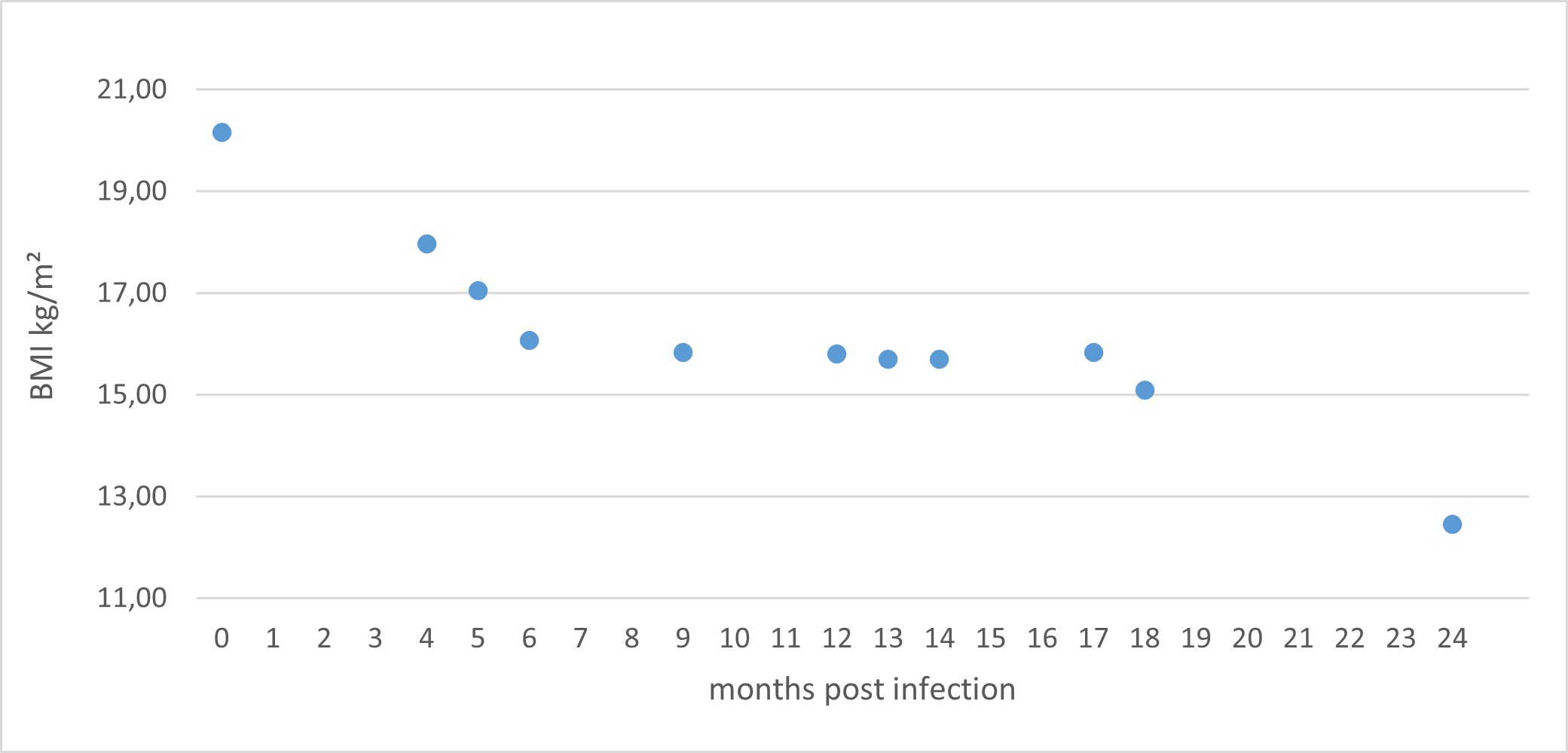

Supplement: SUPPLEMENTARY FIGURE S1A — Course of BMI (kg/m2) over the initial 23 months after EBV infection starting with the premorbid value of 20.1 kg/m2; no measurements available for months 19 to 23; child and adolescent psychiatric inpatient treatment occurred as of month 24 (see Supplementary Figure S1B). [file Image_1.jpg]

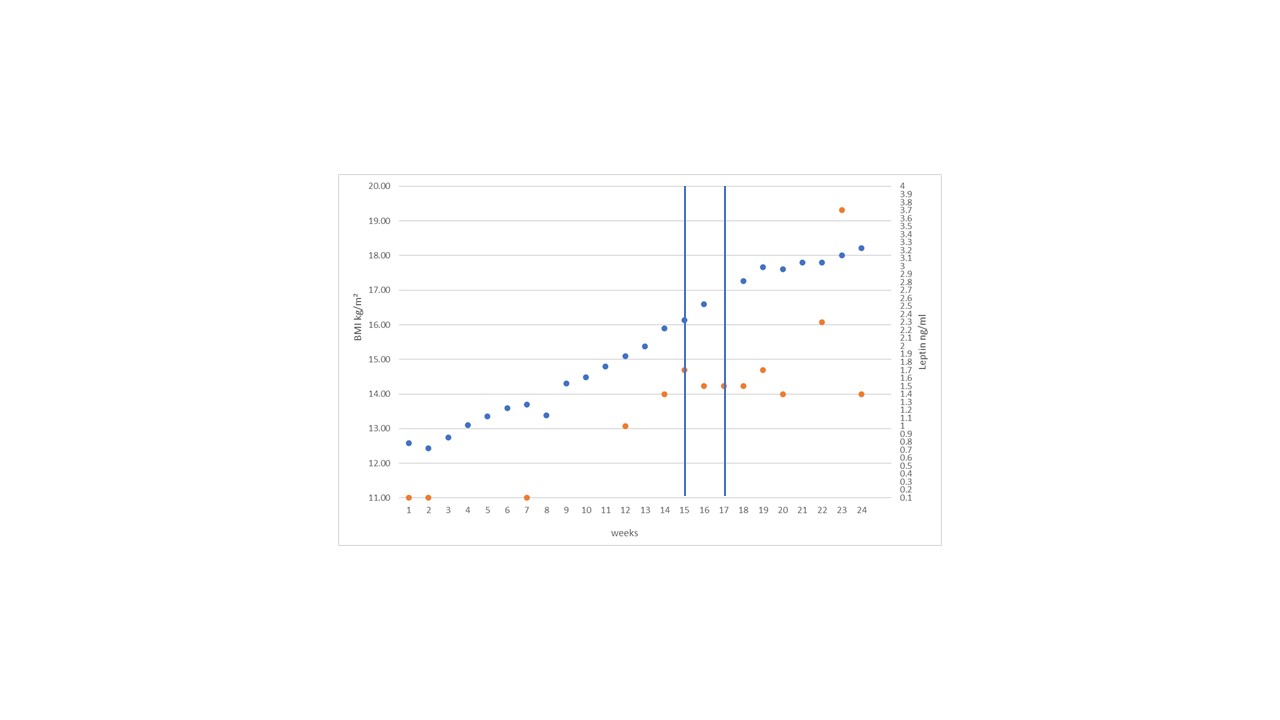

Supplement: SUPPLEMENTARY FIGURE S1B — BMI values prior to, during and after the first 15-day dosing period (marked by the two vertical lines) and serum leptin levels during the 24-week long treatment period. The dosing period was during weeks 15 and 17. Leptin levels weeks 1 to 11 were <0.1 ng/ml. [file Image_2.jpg]

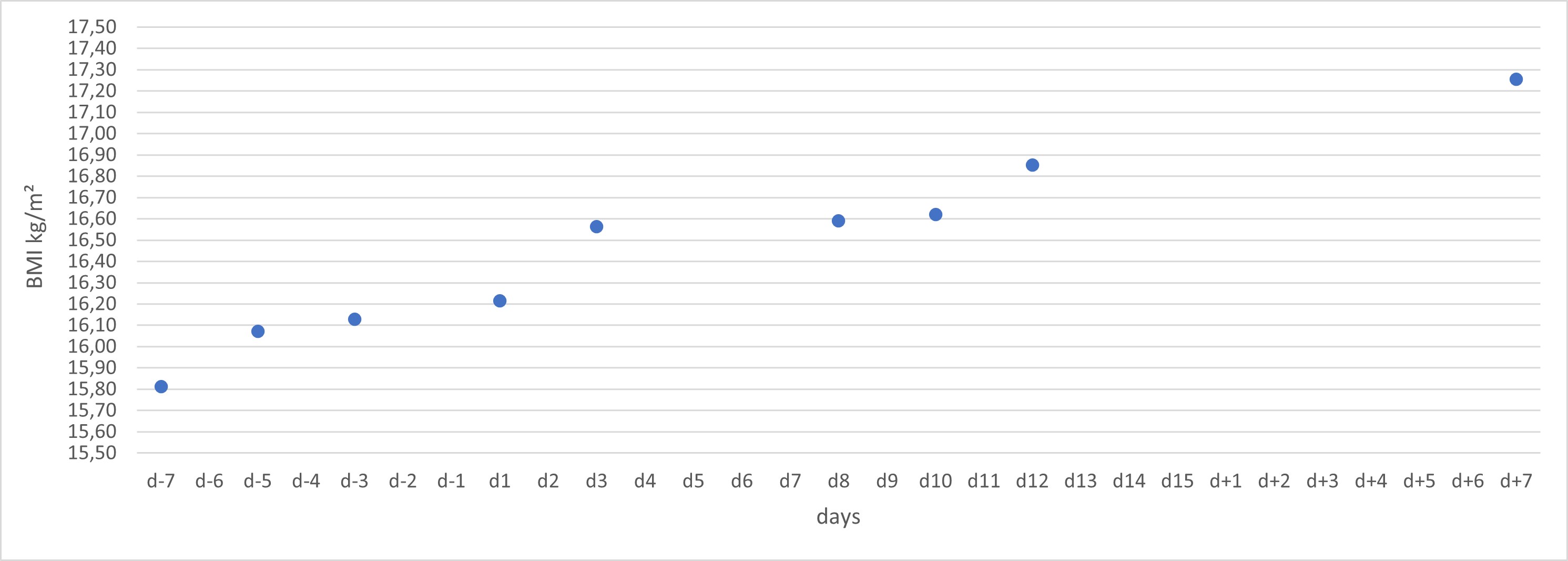

Supplement: SUPPLEMENTARY FIGURE S1B_2 — BMI changes seven days prior, during and after Dosing Period 1. [file Image_3.jpg]

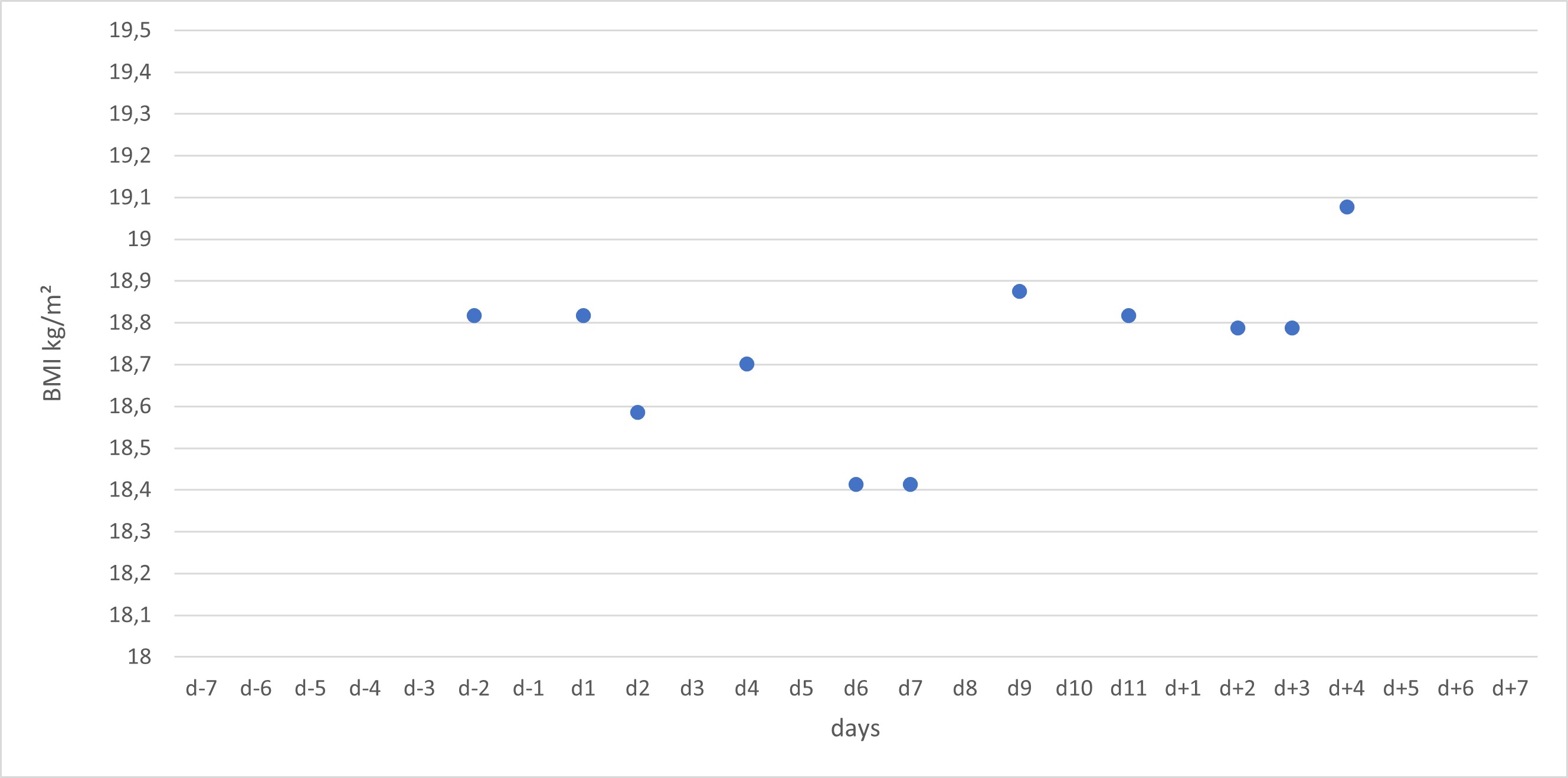

Supplement: SUPPLEMENTARY FIGURE S1C — BMI changes seven days prior, during and after Dosing Period 2. [file Image_4.jpg]

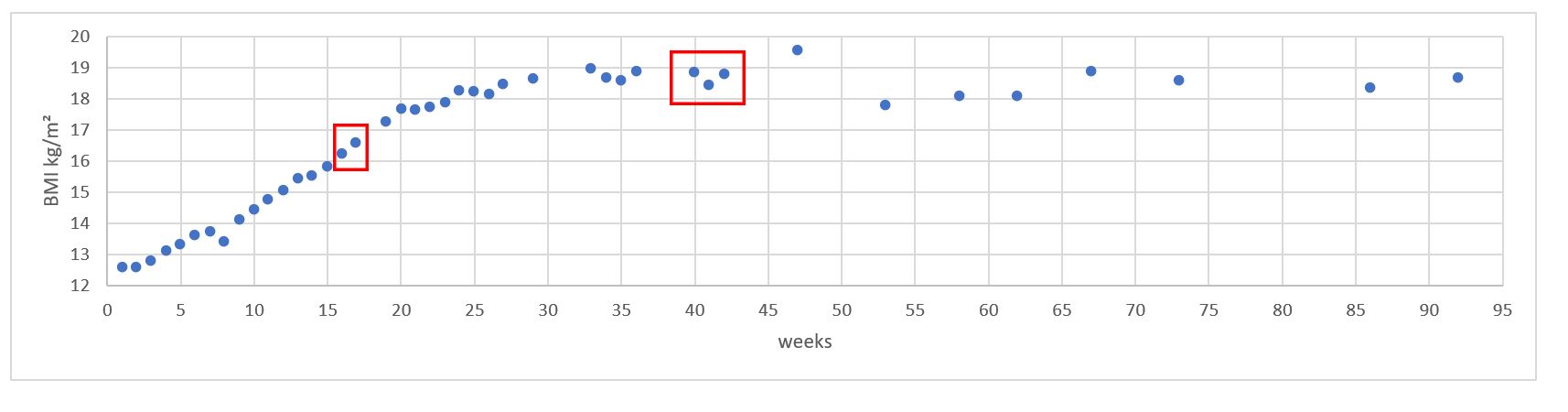

Supplement: SUPPLEMENTARY FIGURE S1D — BMI over the course of treatment; dosing periods marked by squares. [file Image_5.jpg]
